# Magic on the Menu: Where Are All the Magical Food and Beverage Experiences?

**DOI:** 10.3390/foods9030257

**Published:** 2020-02-28

**Authors:** Charles Spence, Jozef Youssef, Gustav Kuhn

**Affiliations:** 1 Crossmodal Research Laboratory, Oxford University, Oxford OX2 6GG, UK; 2 Kitchen Theory, Unit 9A Alston Works, London EN5 4LG, UK; jozef@kitchen-theory.com; 3 Department of Psychology, Goldsmiths, University of London, London SE14 6NW, UK; g.kuhn@gold.ac.uk

**Keywords:** magic, theatrical dining, gastronomy, play, molecular/modernist cuisine, entertainment

## Abstract

Magic and dining have long been popular forms of entertainment. What is more, both involve some kind of transformation, and yet while the more theatrical aspects of dining have grown in popularity in recent decades, there is a surprising paucity of magical food and beverage experiences out there. In this article, we trace the historical appearance of food and drink and culinary items in the performance of magic. We also review some of the more magical elements of food design that have appeared on menus in bars and restaurants in recent years. We introduce the edible lightbulb dish from the menu at Kitchen Theory Chef’s Table and link it to the stage magic of Derren Brown. We also discuss some of the reasons as to why magical food experiences might be rare in the context of dining. In so doing, our hope is to highlight an intriguing area for future research and innovation. Along the way, we identify some possible candidate approaches for the introduction of edible magic onto the menu in the context of modernist cuisine.

## 1. Introduction

Given the long history of magic as a form of popular entertainment [1], and given the public’s growing interest in both playful [2,3] and theatrical food experiences [4,5,6], one would, *a priori*, have imagined that there would be plenty of magical food experiences out there. Indeed, as noted by Hanefors and Mossberg [7], no matter whether or not the restaurant staff are literally magicians, they should nevertheless still be aiming to generate surprise and to play with their diners’ expectations, at least in the context of molecular/modernist cuisine [8]. Meanwhile, from the chef’s perspective, emotional engagement is also key, with the delight/surprise that magic offers chefs providing a wonderful means of immersing guests further into the multisensory dining experience. The more performative and entertaining aspects of magic are, for example, seen at those Japanese *teppanayaki* restaurants where phenomenal kitchen skills and juggling acts contribute to the overall culinary experience [4]. What is more, the evidence suggests that the public’s hunger for magic shows little sign of waning [9].

According to the popular press, several well-known modernist/molecular chefs have been consorting with magicians in recent years in order to optimize the service elements of their dishes [10], or else to present a little magic at the dining table [11]. At the same time, however, what is particularly striking is how few examples of ‘edible magic’ there are out there, at least if one takes a narrow definition of the term. In fact, even the Italian Futurists, who predated many of the most outlandish of dishes/techniques that one finds in modernist/molecular restaurants in recent years [5], surprisingly make no mention of magic [12,13]. Marinetti’s, *The Futurist Cookbook*, for example, does not discuss magic even once [14]. The question that we wish to address here as gastrophysicist, chef, and magician/psychologist, respectively, is whether this absence of examples is just a question of the relevant design work not having been done [15], or whether instead there are some more fundamental reason(s) as to why people might, for whatever reason, find edible magic somehow unpalatable.

## 2. The Psychology and Application of Magic

Conjuring is an art form that allows the viewer to experience the impossible [16]. According to North American magician Teller: *“magic is all about the unwilling suspension of disbelief…[Y]ou experience as real and unreal at the same time…..magic goes straight to the brain: its essence is intellectual.”* [17]. Throughout human history, magicians have developed powerful psychological tricks with which to manipulate our perceptual experiences [18]. That said, magic has received far less attention from those outside the field than most other art forms [19]. In recent years, however, psychological/perception scientists have started to collaborate with magicians in order to uncover some of the psychological mechanisms underpinning a variety of magical illusions [20,21,22,23].

From a psychological perspective, magic elicits a sense of wonder resulting from a cognitive conflict between what you experience (i.e., a rabbit appearing from the hat) and what you believe/know to be possible (i.e., rabbits cannot simply materialize out of thin air; [23]). Neuroimaging evidence supports this notion of cognitive conflict, and further highlights that the conflict experienced when watching a magic trick shares some of the neurological mechanisms that are involved in more general conflict experience and resolution (e.g., [24]). It is, though, important to note that magic can elicit a wide range of other positive (wonder, amazement, surprise, awe, curiosity…) and negative (bafflement, apporea, loss of control…) emotions, which can be embraced by the performer in order to create truly memorable experiences for the audience [25]. A less psychological/neuroreductionist account of magic may be found in Claude Lévi-Straus’s work The scorcerer and his magic, and The effectiveness of symbols, both in [26].

This science of magic allows others to apply these psychological deceptive principles to domains outside/beyond the traditional form of entertainment [16]. For example, some of the positive emotions that can be associated with magic have been used to improve people’s well-being [27]. Furthermore, it has also been suggested that conjuring principles may offer novel means of enhancing people’s gaming experiences [28], and may even provide the means of creating more harmonious human-computer interactions [29].

## 3. Traditional Magic in the Context of Food and Beverage Delivery

It is worth pointing out at the outset here that many live magic performances occur within the context of dining or drinking. Magicians are occasionally employed to entertain guests during drink receptions or to perform magic at the table between courses. This type of magic, known as “walk around magic” or “table hopping”, counts as the bread-and-butter work for most professional magicians [30]. Indeed, it is not uncommon for restaurants to have a resident magician who will entertain the guests with tricks and illusions in order to enhance the latter’s dining experience. The legendary South American restaurant *Andres Carnes de Res* situated in Chía on the outskirts of Bogota, Colombia [31], for instance, has long had various performers, including magicians, opera singers, and comedians, wondering between tables entertaining the festive guests.

At a much smaller scale, *The Twisted Fork* cafe in Stoke Newington, London is run by a member of The Magic Circle who performs while the customers are waiting for their brew [32]. Going to the other extreme, a few decades ago world-famous David Copperfield was planning to open up a chain of restaurants, including one in Disneyland [33] that were to be known as Copperfield’s Magic Underground. The Disneyland venue was to incorporate a themed restaurant together with numerous large-scale magic installations. However, cost overruns and financial/control issues eventually sunk the project which, had it opened as planned in the 1990s, it would perhaps have constituted the most complete merging of magic and dining yet envisioned.

Magicians can sometimes be found working in bars, where the entertainer baffles and amazes patrons with card tricks and other illusions that are performed in very close proximity. In Al Capone’s time, for example, Chicago was well known for its unique form of bar and restaurant magic, inspiring a new genre of magic, known as ‘Chicago Bar magic’ [34]. This involved a combination of humour and magic typically performed from behind the bar in order to encourage consumption. Bar and restaurant magicians often incorporate dining props (e.g., spoons, forks, classes) and food items (e.g., salt, wine) in their routines. At the same time, however, it is important to stress that while the magic occurred in close proximity to those who were eating and drinking, the magical effects themselves were independent of, and separate from, whatever was being consumed. In fact, one might go further and say that in most instances the magicians are essentially oblivious to the food and drinks being served, and the magic is not integrated into the gastronomic multisensory experience in any meaningful sense.

The same is true as far as the magic that has made its way onto the dining tables of high-end restaurants such as *Eleven Madison Park* in New York. There, a card trick was introduced a few years ago as part of the dessert service [11,35]. As Will Guidara, business partner of executive chef Daniel Humm, put it in one interview: ‘We’re not looking to impress people. We want to entertain them’. The magic in this case was designed by Jonathan Bayme and Dan White, illusionists from a company called Theory 11. However, once again, while the magic was performed at the dining tables in this world-famous restaurant, it was not itself edible.

## 4. Using Food and Drink in Magic Performances

Magicians have often incorporated food and drink items into their magic performances. For example, the British comedy magician Tommy Cooper was once famous for producing bottles and glasses from an empty tube [36]. In fact, it is hard to name a culinary item that has not appeared in a magic routine. For instance, the magician Ben Hart performed a beautiful routine on Britain’s Got Talent TV show in which a small piece of paper mysteriously transformed into an egg which is the magician then cracked open into a glass, revealing the yolk [37].

Food, drink, and culinary items are popular magic props, and there are countless ways in which these items can be incorporated into a wide range of distinctive magical effects. However, once again, it is important to stress how most magic tricks simply use these items as visual props. That is, they are themselves rarely incorporated into other sensory illusions. There are, in fact, surprisingly few magic tricks where the audience actually get to eat or drink the magic, or where the tasting experience is itself somehow magical. In fact, perhaps the closest one gets is the Obliging Kettle magic trick, or one of its variants.

### 4.1. Imbibing Magic or Just A Magical Serve?: The Obliging Kettle Trick

On 24 April 1905, magician David Devant presented his new magic show entitled “*A feast of magic*” at St. George’s Hall in London. For one of his tricks, he brought onto stage an ordinary looking tin kettle. Steinmeyer’s [38] description of what happened next is worth quoting at length:

“Picking up a glass, Devant tilted the kettle and poured a small, neat serving of amber-colored liquid.

Is there any gentleman here who happens to know the taste of whisky?

An assistant appeared with a tray and took the whisky to a man in the audience.

Is that good whisky, sir? Good! Now, supposing a policeman, or any other total abstainer, came in and asked the proprietor what they kept in that kettle, he would pour out a glass of cold, white, wet water.

Tipping the kettle, Devant filled the next glass with clear liquid.

Now is there a gentleman who knows the taste of water? I have improved this kettle. It will oblige with any recognized drink you would like to name, ales, wines or spirits. Liqueurs or cordials. Now, don’t shout! Just whisper your orders to the attendants.

Moving to a tray of glasses, Devant poured out dozens of glasses in quick succession: sweet gin, claret, Benadictine, port, and Chartreuse. And the assistants dashed up and down the aisles serving the mysterious drinks, Devant continued with Crême de Menthe and Kummell, finishing with a large glass of milk.”

**Figure 1:**
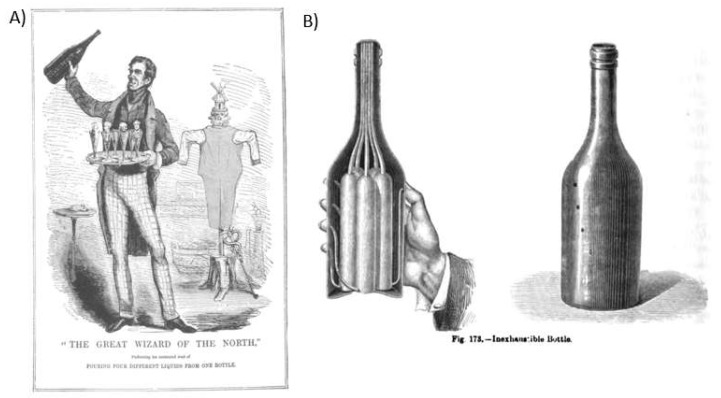
(**A**) John Henry Anderson, the “Wizard of the North” demonstrates the Inexhaustible Bottle trick on the playbill for an 1843 performance at the Victoria Rooms, Hull (UK) from April 1838. [Harry Houdini, re-producing work from the mid 1800’s—[41]; (**B**) Privat-Deschanel’s “Elementary treatise on natural philosophy” explains the use of the Inexhaustible Bottle as a way to cleverly demonstrate the basic principles of hydrostatics (c. 1884) [42].

**Figure 2:**
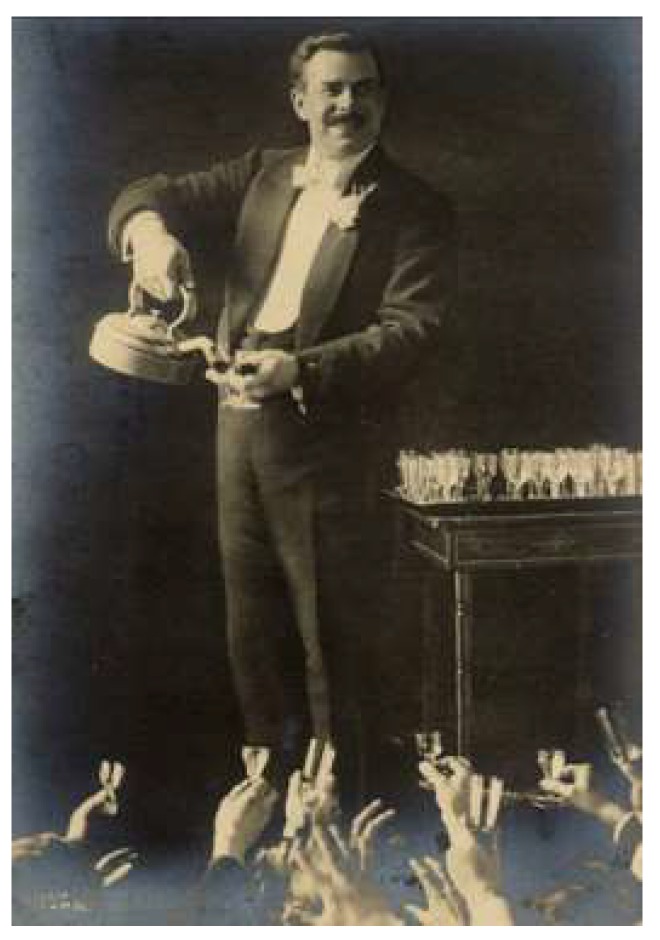
Devant and his Obliging Tea Kettle [36].

The idea of pouring different liquors from a single vessel was already an old trick by the time Devant reintroduced it early in the 20th century. In its earlier incarnation, different liquors were poured from a single bottle, or before that, from a barrel. This magic trick first appeared as the Inexhaustible Bottle in a book from 1635 entitled Hocus pocus junior: The anatomie of legerdemain [39]. Figure 1A shows The Northern wizard performing this trick c. 1843, while the hydrostatic mechanism underlying it is explained in Figure 1B. However, by the time that Devant reintroduced The Obliging Kettle trick to the London stage (Figure 2), it would have been new to most people [38]. Devant himself explained how his version of the trick worked in his book (see [40]). Notice how this is perhaps the first example of magic that is, in some sense, consumed that we have come across so far. That said, the magic here is really in the service element of the drink, rather than in the multisensory tasting experience itself. Given that this trick has once again largely been forgotten by the general public, it might be time for it to be presented anew once again. We believe that it would certainly be interesting to translate it to a bar or restaurant context rather than restricting it to the stage during a magic performance.

This style of magic trick was followed-up very successfully by ‘Think–a-Drink Hoffman’, who can be seen performing his routine on B&W TV show on the following YouTube clip [43]. According to Wikipedia: *“Charles Hoffman, known as "Think-a-Drink Hoffman", performed one of the best known examples as a vaudeville act. His show used a small bar and a series of cocktail shakers which he used to produce any drink the audience asked for, up to eighty different drinks according to his own press materials. During Prohibition, he would produce alcoholic drinks at private shows, and became known as "The Highest Paid Bartender in the World".”* [44,45].

### 4.2. Cocktail Magic

Historically-speaking, playfulness has tended to be more apparent in the world of cocktails than in the most outré of modernist/molecular food creations [46]. One example of magical cocktail experience was the Illusionist menu at Bar Paradiso in Barcelona. Take the following description of the magical experience from The World’s 50 Best Bars: *“Take a bow, the Illusionist menu. Housed in vessels (if we can call them that) bespoke-made by local craftspeople, some cocktails change colour, others transition from sweet to bitter, there are those that are guillotined in half in front of you and others you can’t even find. But if drinks in Trojan Horses aren’t your thing, go for the classic twist Super Cool Martini. Made using a proprietary technique, the mixing of the liquids sees an iceberg freeze in the glass. Just another bit of the unexpected from the Paradiso box of tricks.”* [47]. The Beaufort Bar at *The Savoy* in London has also introduced a new magic-themed cocktail menu recently too [48] (interestingly, not everyone thinks that putting magic on the menu is interesting currently. Hamish Smith, editor of one of the big cocktail magazines here in the UK had the following to say: *“… in the UK, the theatrical/magic side of cocktails is seen as a little old hat. The belief being, for liquids to be considered as important as solids, drinks need to be about flavour and ingredients, not tricks”* [49].

A colour-changing cocktail also appeared on the menu at the Harry Potter-themed bar in London/New York [50,51]. Within the field of dining, the term ‘food magic’ seems restricted to chemical reactions like changes in PH, as in colour-changing cocktails that use pea flower [52]. However, looking to the future, it may be possible to use a range of different techniques to elicit transformation, such as a change in colour, thus leaving the diner/drinker uncertain as to the explanation for the transformation that they have just experienced: Is it a chemical transformation, a psychological/perceptual illusion, or some form of mentalism [51,53]?

### 4.3. Aromatic Magic

Another example of magic involving chemosensory stimuli is an illusion that involves olfactory stimulation. The magician Robert-Houdin performed a beautiful levitation effect known as the ethereal suspension. He told his audience that ether has a magical property of making people levitate. He took a small bottle and opened it under his son’s nose. The audience could smell the ether as it wafted through the theatre. In reality, the bottle was empty and instead the real smell came from his stagehands pouring ether onto a hot shovel. Along similar lines, Slosson [54] performed a smell hallucination study with his class of students that bears some similarity here. The professor opened up a bottle at the front of the class, telling his students that the smell would slowly diffuse throughout the auditorium and that they should lift their hands when they smelled it. Slosson describes how the students at the front of the class started raising their hands and complaining about the smell, followed by those in rows that were further back. In fact, the bottle was empty. This laboratory demonstration therefore nicely illustrates how suggestible people are in terms of phantom smells.

More recently, O’Mahony [55] demonstrated a very similar effect on television and radio audiences. During a television program on the chemical senses on Granada Television in the UK, the audience heard a tone, which they were told would cause them to experience a pleasant country smell. Many viewers wrote in reporting that they had perceived the smell of grass or hay. Several people even wrote in to complain that they had suffered from attacks of hay fever and sneezing after listening to the tone! Once again, the belief that an odour was present was enough to trigger symptoms in many people, even though there was, in fact, no specific odour present. Several people listening to a BBC Radio Bristol show even reported olfactory sensations when an “ultrasonic tone” (actually silence) was played across the airwaves!

## 5. Historical Examples of Magical Food Presentation

Switching themes a little, a number of putatively magical looking food-presentations described have been in the past. One might, for example consider the wonder that must have been experienced by diners in ancient Rome on first seeing the colour-changing surmullet fish paraded around in front of guests [56]. The skin of this fish would magically transform spectacularly through a rainbow of colours. Also relevant here is the historic grand feasts where roast animals would be carved at the table only for the guest to be amazed when live animals suddenly emerged from the inside [57,58]. One other visual trick in such feasts involved sewing the front-end of one animal on to the back end of another to create mystical beasts for the assembled guests to consume.

### 5.1. Animate Foods

In terms of such grotesquely-magical dishes, one might also consider the much more recent popularity of dancing-squid-type dishes in parts of Asia, where dead creatures that miraculously appear to come back to life [59]. There is both something disconcerting and at the same time-attention capturing and Instagram-worthy about such foods (reminiscent in a way of rubbernecking drivers straining to see the carnage on the other side of the carriageway). This kind of ‘re-animation’ could perhaps appear magical, though rarely is it framed as such by the popular press. Katsuobushi flakes provide another culinary element (typically experienced in the context of Japanese cuisine) that moves dynamically on the plate due to the heat and creates an almost magical experience for those who are unfamiliar with it.

### 5.2. Magical Food on the Fairground

One of the places where it might have been expected that edible magic would have emerged was on the fairground, given that both food and close-up/stage-based illusions would have been on offer to the punters. In fact, the first author’s great, great grandfather Randall Williams, the self-proclaimed King of Showmen, toured with illusions such as the phantasmagoria and Dr. Pepper’s Ghost in the closing decades of the 19th Century in the North of England fairground circuit. Why did no enterprising soul consider putting these two together, especially since food items, such as coconuts were integrated into other elements of the fairground entertainments, think here only of the coconut shy [60,61] (notice here only how the ice-cream cone apparently came about as a result of the proximity of ice-cream and waffle stalls at the 1904 St. Louis World’s Fair in North America). That said, according to the historical record, whilst the magicians would have appeared at the statutory fairs that took place across the UK, the magicians themselves tended to keep themselves apart from the fairground community [62], thus presumably reducing the opportunity for interaction.

## 6. Magical Service Experience in the Restaurant Context

Much of the magic at the dining table these days turns out, on closer inspection, to reside in the service element of the dish. It is these examples that we review first. These examples can be seen as bringing the magic and food/drink closer together than may previously have been the case without ever quite delivering a magical multisensory tasting experience.

### 6.1. Levitating Food/Serviceware

**Figure 3:**
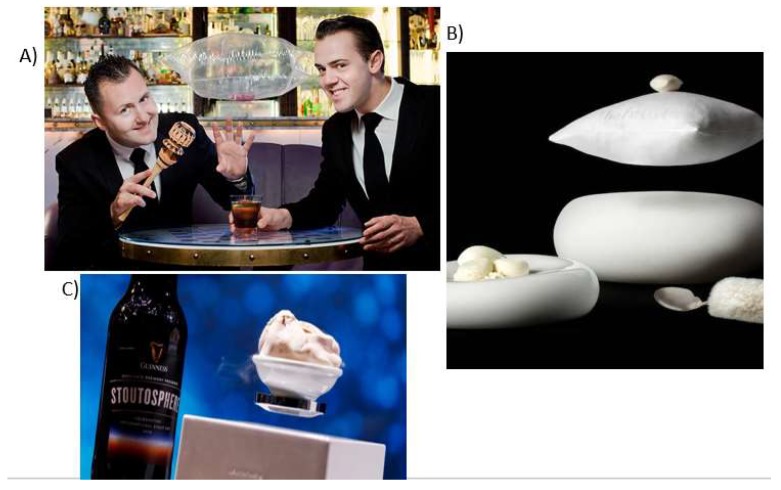
Three examples of levitating drinks/dishes. (**A**) *Bar Artesian* in London; (**B**) *The Fat Duck* floating pillow *Counting Sheep* dessert, in which a meringue rests on a pillow that floats and spins six inches above the table [63]; (**C**) *Kitchen Theory* levitating ice cream [Photo credit: John Carey]. Notice how in all three cases while the service element of the drink/dish is in some sense magical, the tasting experience itself is not.

Levitation has been used successfully in recent years in the context of both bar cocktails and desserts in the context of the fine-dining restaurant. Figure 3 shows a trio of popular examples of levitation from the UK. Note, though that while the service elements are, in some sense magical, the tasting experience itself is not.

### 6.2. Magic at the Dining Table

Meanwhile, a few years ago, world-leading chef Heston Blumenthal conferred with magicians while experimenting with a flaming sorbet that was designed to ignite at the click of the waiter’s fingers. According to one journalist: *‘Blumenthal created it with a magician so, at the click of a waiter’s fingers, the barley sorbet in a bowl of hidden compartments bursts alight, turning warm outside yet remaining ice-cold inside. As a fire crackles around the sorbet, a rolling vapour of whisky and leather transports you to some Scottish hunting lodge at Christmas.’* [64]. While the idea sounds intriguing, it is worth noting that the magic element in this case concerns the service elements of the dish rather than the flavour experience itself. The bowls were rumoured to have cost £1000-a-piece perhaps explaining why this dish did not, at least not as far as we are aware, really ever make it into mainstream service.

### 6.3. A Magic Trick to Reveal the Petit Fours

Another example of magical service was presented with the *petit fours* at Jozef Youssef’s *Kitchen Theory* [65] a couple of years ago. The diners were presented in a small wooden box locked with a three-digit roll lock. As the boxes were placed down in front of the expectant diners in the single sitting service, the chef would come out from the kitchen. He encouraged those assembled to think of a number between one and nine, to double it, add 8, divide by 2, and subtract the original number you were thinking of. Low-and-behold the lock ‘magically’ opens to reveal the *petit fours* (the result is always four). This simple number trick was sufficient to amaze many of the diners, while motivating other more mathematically-minded souls to try to figure out how it was done. Once again, though, note how the magical element is in the service not the food itself.

## 7. Edible Magic

Having described the various examples of magical service, we can now focus on those examples of what can genuinely be described as edible magic.

### 7.1. Disconfirmed Expectation: Illusion on the Plate

Modernist/molecular cooking has certainly provided imaginative chefs with the opportunity to play with ingredients and deconstruct dishes in ways that many diners find surprising, at least initially [8,66,67]. Just take The Roca Brothers white-coloured dark-chocolate ice-cream, or Heston Blumenthal’s Beetroot and Orange Jelly, for the playful/surprising disconnect between the colour or visual appearance and flavour in a dish. However, it is worth pointing out here that disconfirmed expectation, at least as far as flavour is concerned, is challenging to pull off successfully without giving rise to a negatively-valenced ‘disconfirmation of expectation’ response [68,69,70,71].

One other intriguing dish that involves an element of illusion is the Nitro-scrambled egg and bacon ice-cream, as served at Heston Blumenthal’s *The Fat Duck* restaurant [72]. The contents of an eggshell are removed by making a tiny hole in the shell. An ice-cream mixture is prepared and the latter is then surreptitiously injected back into the eggshell using a syringe. A primus type stove and frying pan are wheeled to the table. From the diner’s perspective, it looks as though a raw egg is cracked into the pan, and then the mixture is cooked in liquid nitrogen. The dish also delivers a satisfying sonic crackle, a little reminder of sizzling bacon, perhaps. The illusion is created that a raw egg can be turned into bacon and egg-flavoured ice-cream.

**Figure 4:**
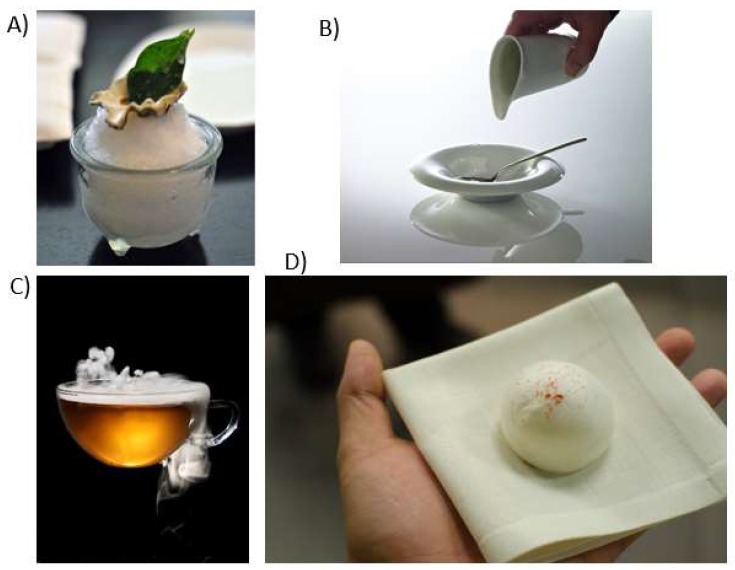
(**A**) Oyster leaf served as one of the courses at Grant Achatz’s *Alinea* restaurant in Chicago. This leaf tastes just like oyster; Diners may be left wondering ‘how on earth did the kitchen team manage to do that?’ while in fact, the leaf grows naturally on the shoreline (e.g., in Scotland; (**B**) ‘Rien’ the mysterious invisible dish by Denis Martin from the 2-Michelin starred namesake restaurant [74]; (**C**) The hot and iced tea as served at Heston Blumenthal’s *The Fat Duck* restaurant ([75]); (**D**) The ‘rattle’ from Mugaritz (c. 2014). But where does the noise come from?

There has long been interest in tromp l’oeil in food design ([73], for recent discussion; trompe-l’œil is an art technique that uses realistic imagery to create the visual illusion that what is depicted really exist in three dimensions). There are also ingredients that taste of one thing while looking like another. Think here only of the oyster leaf, also known as vegetal oysters. These green leaves, which look very similar to a sage leaf taste, for all the world, like oysters. Presenting a leaf as a dish has been used by some chefs (e.g., Grant Achatz at *Alinea*; see Figure 4A). Diners may be mystified asking themselves: *‘How on Earth did they do that?’* But the intuition is that there was a culinary process behind the surprising tasting experience, and that a trip to the kitchens would likely reveal all. The magic, one might say, is more in the kitchen, or at least is assumed to be, by the diner.

### 7.2. “Rien”: Where’s the Food?

Another candidate for a magical dish comes from Denis Martin’s two Michelin-starred namesake restaurant in Vevey, Switzerland [74] where the chef once served a dish by the name of ‘*Rien*’ (which means ‘nothing’ in English). An empty bowl with a spoon resting inside would be brought to the table (see Figure 4B) with the waiter mischievously saying *“Just a little something?”*. As a diner, you end up looking at the empty bowl sitting in front of you wondering what to do. The waiter then proceeds to pour nothing from a sauceboat into the bowl with a conspiratorial air. Once your plate is filled with ‘nothing’, you are left puzzled. “You do not taste your food?” the waiter might ask. Not knowing what else to do, you try bringing the empty spoon up to your lips to mimic the act of eating. And then, surprise, surprise, an intense burst of flavour suddenly fills your mouth: tomatoes, cucumbers, *gazpacho*… The spoon has been coated with a transparent film of a very tasty invisible flavour that is, in a sense, magical. “How on Earth did they do that?” the diner might be left wondering.

### 7.3. Hot and Iced Tea

A magical drink served for a time at *The Fat Duck* was the ‘Hot and Iced Tea’ (see Figure 4C). The drink, served in a transparent glass, was hot when first tasted by the diner. However, when the cup was rotated 180 degrees the drink was suddenly ice-cold. Diners are often perplexed as to why the tea, which is experienced at different temperatures, do not mix in the glass giving rise to a liquid that is uniformly tepid. The reason for this seeming physical impossibility is that the tea itself is actually made of a very finely chopped gel that just behaves like a liquid; hence the hot and cold parts resist mixing. To make the dish, a barrier is initially placed in an empty thermally-insulated glass, and then the hot gel is poured in one side, the cold gel in the other. Next, the barrier is removed, and the dish promptly presented to the expectant diner. A similar technique was first used by Ferran Adrià back in the early 1990s when he served ‘Hot and Cold Pea Soup’, one of his early signature dishes. Served in a shot glass, the soup was hot on top and cold on the bottom.

### 7.4. The Mugaritz Rattle

Another food experience that might count as edible magic was presented at chef Andoni’s *Mugaritz* restaurant on the 2014 menu (see Figure 4D). It was described as an edible rattle that made a noise when shaken, as if there was a ball-bearing inside. According to one food blogger:*“…we were given a “rattle” to eat — shake it and it makes rattling sounds. Full of togarashi, caramelized onions and magic.”* [76]. The ‘magic’ in this case, at least according to those colleagues who have had the opportunity to try the noisy morsel, was that when consumed in one bite there was no sound-making element inside. The diner is once again left wondering “How on Earth did they do that?” However, since the sound-emitting morsel has already disappeared into the diner’s mouth, there is no way of investigating. And thus, the attentive diner is simply left to ponder.

### 7.5. Edible Magic: When Magician Meets Modernist Chef

**Figure 5:**
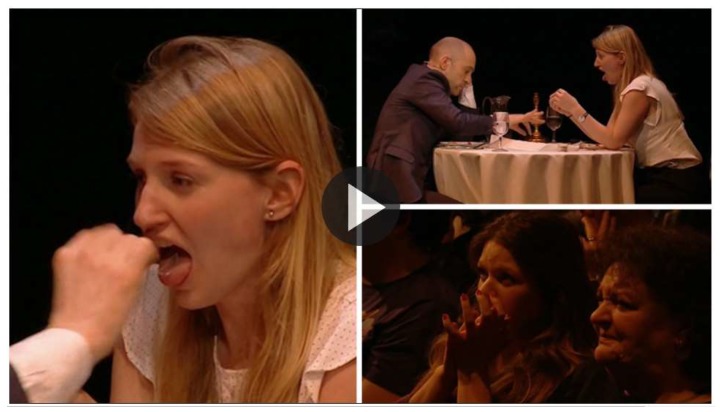
Fear on the face of guests as Derren Brown magically gets them to eat what looks like a shard of glass from a broken light bulb. Is one of the reasons that magical food experiences are so rare, the fact that so many examples of magic in mouth are gruesome/unpleasant. The fear is palpable on the face of the audience [77].

The British magician Derren Brown caused a stir in 2016 when he got an audience member to eat what looked like a piece of broken glass from a light bulb in front of a TV audience (see Figure 5) [77]. In Brown’s hands, the audience member was encouraged to carefully pick up a shard of glass with a napkin (meaning that the latter is unaware of the slightly sticky surface texture), and bite into it. Having done so, they were encouraged to bite a slice of apple, presumably masking the sweet taste of the sugar in the glass candy.

**Figure 6:**
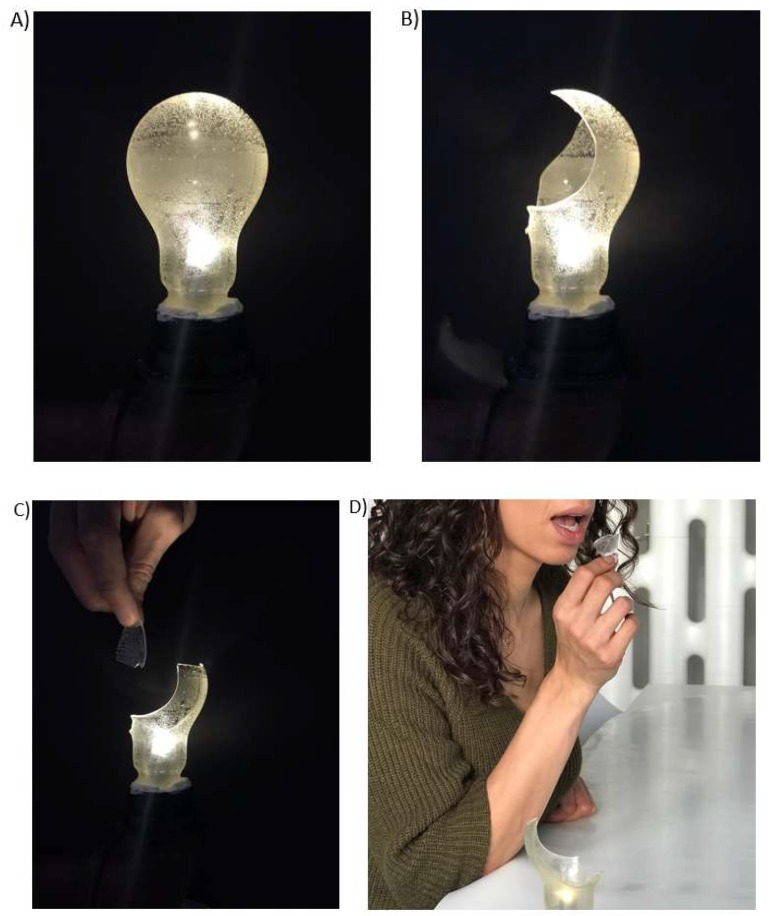
The edible lightbulb dish as served at *Kitchen Theory’s* Gastrophysics Chef’s Table. (**A**–**D**) from presentation to consumption.

Intriguingly, a version of this trick has now been incorporated onto the menu at *Kitchen Theory* (see Figure 6). This example of edible magic is the only one that we are aware of that has been performed both on the magic show stage (or TV show) and in the restaurant setting. The recipe for the dish can be found in the Appendix A. While this can, we believe, justly be given as an example of edible magic, it is interesting that the flavour experience itself is not magical, and the illusion is really about the texture of a seemingly inedible object.

### 7.6. The Magical Completion of a Dish at Table

Albert Adriá has also created a dish that is prepared table-side where a waiter pretends to combine random ingredients in a pot or bowl. To the diner, it looks pretty unlikely that the process will result in a finished dish. The mess is covered with a ‘magic lid’ which, when it is removed again, reveals a finished course right in front of the diner’s amazed eyes. Although the dish is apparently only served to children and friends, one catches a glimpse of this in the first 10 minutes of the Albert Adria episode on Netflix’s successful series *Chef’s Table*.

## 8. Edible Magic: Lost Opportunity or Unpalatable Experience

Having highlighted the relative rarity of magical food experiences, or magic that you can eat or drink, this raises the question of whether such experiences might represent a natural direction for modernist gastronomy to move in, especially given the growing interest in theatrical dining and the merging of different forms of entertainment—think here only of Secret Cinema-type events where the audience get to eat foods linked to the action on the screen [5,6]. Relevant here, there are several magical flavour-related phenomena that have been reported in laboratory research that might potentially be worked into a magical dish or drink experience in the near future.

## 9. Laboratory-Based Flavour Illusions

### 9.1. Oral Referral

The one demonstration from the science of flavour perception that regularly elicits an audible groan of wonder from the members of the audience comes from the jellybean test. This long-standing dinner-party illusion [78] involves people being told to hold their nose shut with their fingers and to bite into a jellybean. They will only be able to perceive sweetness and possibly a little sourness. However, as soon as they let go of their nose, they suddenly experience a burst of fruity flavour resulting from the transduction of the retronasal olfactory cues [79]. One often hears an audible ‘Ahhh’ spreading around the room. The surprise that this demonstration elicits in people can then, in some sense, be seen as equivalent to what one sees in a magic trick.

And yet, the fact that the surprising experience is localized within the confines of one’s own body, again makes it seem to occur outside the space where magic normally operates. It is almost as if people reason that whatever happens within themselves must be under their own control, and hence beyond the remit of magical interference.

### 9.2. Choice Blindness in the Marketplace

Choice blindness is an intriguing sleight-of-hand trick with interesting psychological consequences [80]. In the original version of this phenomenon, people were shown two faces on cards, and asked to rate who they found more attractive. The task is repeated with several different pairs of cards. Then, on one trial after having made a choice, the experimenter would apparently turn over the card to reveal the chosen face again, and ask the participant to justify why they had selected that one. Many participants fail to spot that the card they are been shown the second time round is actually the face of the person that they just rejected. Nevertheless, many people then go on to justify why they chose the face that they had not, in fact, chosen a moment before. One of the intriguing aspects of the original choice blindness experiment is that it works better with an experimenter, rather when the participant sees the cards being turned over on a computer screen, say.

In a follow-up study using food stimuli, double-ended pots of jam were used [81]. As demonstrated on an episode of the Horizon TV show (UK), customers in a supermarket got to taste two different jams/teas and were asked which one they preferred. Once again, after having made their choice, the shoppers were invited to taste their preferred choice a second time and explain their decision. Unbeknownst to the participants, the double-ended jam jars had been inverted, and the second time they are justifying the choice for the taste of the product that they did not pick first time around (it is worth noting here that the flavours of the jam and of tea need to be reasonably similar in order for this trick to work). This then counts as a sleight of hand involving food stimuli. And yet at the same time, while a large proportion of people are fooled by this trick, the deception does not really count as magic as the taster themselves typically remains unaware that anything is amiss. The deception only becomes apparent when the trickery is explained. As Darwin Ortiz, a well-known magician, once put it: *“Magic is not simply about deceiving. It’s about creating an illusion, the illusion of impossibility”* [82].

### 9.3. The Butcher’s Tongue Illusion

In some of our more bizarre food-related laboratory research at the Crossmodal Research Laboratory here at Oxford University, we have mislocalized taste out of the mouth and on to a butcher’s tongue (or a rubber imitation of a human tongue—For those who are familiar with it, this a version of the famous rubber hand illusion [83,84]). The participants extend their tongue in a mirror box. They see a butcher’s tongue in an anatomically appropriate orientation via the mirror arrangement. The experimenter then gently strokes both the participant’s tongue (out of sight), in synchrony with the butcher’s tongue. A number of participants take ownership of the butcher’s tongue (i.e., they become convinced that they can see their own tongue). Then, when a cut lemon is, say, squeezed on the butcher’s tongue, while water is dropped on the participant’s own tongue, some have been convinced that they can taste lemon juice [85]. While this really might count as a kind of oral magic, the trick has yet to make it from lab and science festival to dining room (one US magician who was filming a show tried the Butcher’s tongue illusion and was heard to exclaim: *“Now, that really is magic.”*).

### 9.4. Magic Potions and Miracle Pills

In folklore and fiction, one finds the notion of the magic potion. In *Alice in Wonderland*, for example, Alice is offered the pill that will make her bigger or smaller [86]. Indeed, some of the more magical elements found in this classic tale have provided inspiration for Heston Blumenthal’s culinary creations at The Fat Duck restaurant [64]. Here, though, the thing that is consumed is not itself a magical experience, but is merely the vehicle via which to get to the magical experiences. Linked with the notion of consuming in order for something magical to occur, some modernist chefs have served the aptly-named miracle berry pills to their guests for dessert. These pills, made of miraculin extracted from the fruit of a West African tree give rise to a miraculous effect when rolled around the tongue and mouth, namely subsequently-experienced sour foods, like a slice of lemon suddenly start to taste sweet [87,88,89].

However, while the transformative effect in taste induced by miraculin, the active ingredient could, in principle, be magical (if, say, its administration to the taste buds was achieved surreptitiously, by say, coating a lollipop with miraculin and then getting the diners to lick it, the way in which the pill is typically administered tends to take any mystery from the experience. Many diners are undoubtedly mystified as to how it is the slice of lemon tastes sweet, but at the same time they can correctly ascertain that it must be some sort of chemical reaction with their taste-buds that they have not come across before. Here, one is reminded of Simon Aronson’s comment that: *“There is a world of difference between a spectator’s not knowing how something’s done versus his knowing that it can’t be done.”* [90,91]. What is more, the long-lasting nature of the change in taste experience, lasting for 45 minutes or so, also makes it difficult to manage from a magic trick perspective. Here, the fact that once food enters the mouth it becomes a very personal experience, one that is not shared (here, one might also consider *Space Dust*/Popping Candy as a kind of magical food, at least amongst children [92], and one could also mention *Lucky Charms* as a children’s food product that is in some sense marketed on the basis of magic), and yet, the fact that the surprising experience is localized within the confines of one’s own body, again makes it seem to occur outside the space where magic normally operates. It is almost as if people reason that whatever happens within themselves must be under their own control, and hence beyond the remit of magical interference.

## 10. Future Directions—How Might the Art of Magic Complement Our Food Experience?

In this final section, we discuss possible ways in which the art of entertainment magic could potentially enhance the culinary experience. Magicians use misdirection and psychological tricks to create the illusion of impossibility, and some of these principles could be incorporated into the dining environment to create a new form of olfactory and taste experience. One way of doing so would be to use deception and trickery to alter people’s taste sensations. In The official CIA manual of trickery and deception, magician John Mulholland describes numerous deceptive tricks that allow special agents to secretly add poisons and other substances into their enemies’ drinks [93]. Similar techniques are often used by stage hypnotists to surreptitiously add vinegar to water to enhance hypnotic taste suggestions. We obviously do not wish to suggest that chefs or cocktail makers poison their guests, but a playful use of this sort of illusion and deception could potentially be deployed to help create some perplexing and magical taste experiences (cf. the Hot and iced tea, mentioned earlier).

Magic allows us to experience the impossible, and there are countless illusions and deceptive tricks that can potentially be used to create truly magical dining experiences. Some of these tricks may simply amuse, but they may also alter the diner’s taste sensation in ways that are playful. For example, a multitude of deceptive techniques that can be deployed to surreptitiously switch items, or visibly transform them in front of the spectators’ eyes. An olive could transform into a strawberry, or water could literally be turned into wine (in passing, one should probably also mention the miraculous "Feeding of the 5,000" that appears in all four gospels in the Bible—Matthew, Mark, Luke, and John. According to the relevant Wikipedia entry: *“Taking the five loaves and the two fishes and looking up to Heaven, he gave thanks and broke them. Then he gave them to the disciples, and the disciples gave them to the people. They all ate and were satisfied, and the disciples picked up twelve baskets full of broken pieces that were left over. The number of those who ate was about five thousand men, beside women and children”* [94]). Some of these techniques rely on skillful sleight of hand, but there are many other more self-working techniques that could be deployed at the dining table, even by chefs and serving staff who have not had much practice of performing such tricks. At the same time, it should also be borne in mind that the cost associated with having a trained person/magician individually performing magic tricks tableside may be too much for all but the high-end establishments.

Magic allows us to alter the laws of physics, allowing one to create engaging and enjoyable sensory experiences. Imagine a cloud of edible candyfloss mysteriously floating across the table into the diner’s mouths—this might provide a new dimension to a light and fluffy meal (cf. [95] for a system that can deliver floating droplets of solution to a taster’s tongue). Food items could appear from nowhere. These types of effects are often performed for children (e.g., producing sweets from behind the child’s ear), but such magical manifestations might indeed transfer a form of magical essence to the object (i.e., sympathetic magic). The experience of the magician amongst us authors suggests that people often treasure mundane objects that have undergone some form of magical transformation (e.g., a piece of rope that has been magically cut and restored; see also [30]), and such magical transformation could add new sensory dimensions to the dining experience.

Not all magic relies on sensory illusions. Mentalism is a form of magic in which the magician reads your mind and predicts the future [16]. This is one of the most popular forms of magic, and aspects of these types of illusions could easily be incorporated into the dining experience. Imagine a restaurant in which orders are transmitted telepathically. A waiter who serves the correct drink even before it has been ordered. Such mind games and illusions may not necessarily change people’s sensory experiences, but they would certainly add a layer of mystery and excitement to the presentation of dinner; this approach can also be seen as linking in with the increasingly popular notion of personalization [5].

## 11. Why Magical Food Experiences Might not Be the Thing

At this point, one might wonder whether the reason for the rarity of magical food experiences reflects nothing more than a lack of imagination on the part of chefs/magicians, or whether instead there is a more fundamental reason as to why such an approach might be destined not to succeed. While one might argue that the delivery of food to the audience requires close-up contact and so means that this is not really a possibility for large stage magicians, it is worth remembering that the Obliging Jug illusion was performed in a large stage setting. What is more, the close-up contact is actually ideal for using magic in a dining environment, as illustrated by Derren Brown’s shattered glass eating trick. It is certainly one of the most popular and most innovative magic genres. However, there are enough close-up magicians and that is presumably the domain where such magical food experiences could be crafted.

Another possible explanation for the lack of edible magic concerns the danger of poisoning. The psychologist Paul Rozin has also written extensively about danger as an important reason as to why people might refuse to eat certain foods [96,97,98]. After all, according to some researchers, while we are happy to be fooled by what we see/hear, as soon as something enters our mouth then that is the last stage before we are potentially poisoned. As such, being tricked or surprised about taste may not be such a pleasant experience. This was the explanation that Debra Zellner and her colleagues came up with to try and explain why the use of inappropriate colour might lead to visual dominance over orthonasal but not over retronasal olfactory judgments [99]. Along similar lines, Zampini and Spence [100] also reported that while modifying the sounds of carbonated drinks biased their participants’ perception of how carbonated drinks were when visually inspected in a plastic cup, no such crossmodal influences were observed once the participants held the drink in their mouths. The suggestion here being that crossmodal illusions/sensory dominance do(es) not occur so often once stimuli enter the mouth.

Another point to note here when thinking about the senses and magic is that the vast majority of magic tends to be visual [101,102,103,104,105]. Visual dominance is widespread across many fields of human endeavour [106,107], and it might be argued that the meta-cognition that is such a key component of magic tricks is simply more developed in vision than in our other senses.

There are simply far fewer magic tricks in the auditory modality (excepting perhaps the Shepherd staircase), the tactile modality, or the olfactory modality, though these have been a few illusions that occur only when people cannot touch what they see [108,109]. What is more, those illusions that involve more than one sense are typically grounded by visual input, as in the ventriloquism effect [108,110]). There is, in other words, a profound visual dominance and, as such, food primarily stimulating, as it does, the chemical senses does not necessarily fit, though the tromp l’oeil examples mentioned earlier do fit in this space.

One other consideration here is that information processing in the chemical senses might simply not be as fast [111], or as content-rich, to afford the kinds of errors that give rise to magic. This likely explains why perceptual switching appears to occur more regularly in vision and audition say, than it does in terms of flavour perception [112].

## 12. Conclusions

As this brief review has attempted to make clear, there is a surprising lack of food or beverage experiences that can legitimately be described as edible magic. More theatrical and entertaining food experiences that are have become increasingly popular in recent years [5,6]. The suggestion that it is OK to play with your food has also become much more widespread [2]. At the same time, however, genuinely magical food and drink concoctions turn out to be surprisingly rare. Nevertheless, the addition of magical elements to service is becoming an increasingly popular endeavor. In this review, we outline a small number of magical tasting experiences that have appeared in the context of the magic show or restaurant. We also provide the recipe for the edible lightbulb illusion, a version of which was first introduced by magician Derren Brown [77].

Several possible reasons as to why people might not enjoy edible magic are discussed, including the fact that most magic tends to be visual, and the fact that as the last gate before something might poison us, tricky may be less acceptable. Looking to the future, our hope is that it may be possible to create a dish, where the diner/drinker is left perplexed as to the source of the illusion: Was it culinary magic, some kind of psychological illusion, or perhaps some form of mentalism (a la Derren Brown)? Of course, should it be possible to create such edible magic illusions that still leaves us with the question of where the natural home for such trickery would be: Magic show or modernist restaurant?

## Appendix A. Edible Lightbulb Recipe

Ingredients: 500 g isomalt; 50 g distilled water.

Methods: In a pot, add the distilled water to the isomalt and stir until evenly distributed and the mixture has a ‘wet sand’ consistency. Place the pot over a high heat and use a silicone spatula to move the mix. Using a sugar thermometer bring the mix up to 167 °C. Grease the 3D silicone light bulb mould. Pour in the mix from the pan in to the silicone lightbulb mould, then flip upside down over a Pyrex dish so excess isomalt pours out. Allow to cool for 15 minutes then open the mould and remove the lightbulb shell.
